# Eriocalyxin B Inhibits STAT3 Signaling by Covalently Targeting STAT3 and Blocking Phosphorylation and Activation of STAT3

**DOI:** 10.1371/journal.pone.0128406

**Published:** 2015-05-26

**Authors:** Xiaokui Yu, Li He, Peng Cao, Qiang Yu

**Affiliations:** 1 Department of Pharmacology, Shanghai Institute of Materia Medica, Chinese Academy of Sciences, Shanghai, China; 2 Laboratory of Cellular and Molecular Biology, Jiangsu Province Academy of Traditional Chinese Medicine, Nanjing, China; Columbia University, UNITED STATES

## Abstract

Activated STAT3 plays an important role in oncogenesis by stimulating cell proliferation and resisting apoptosis. STAT3 therefore is an attractive target for cancer therapy. We have screened a traditional Chinese herb medicine compound library and found Eriocalyxin B (EB), a diterpenoid from Isodon eriocalyx, as a specific inhibitor of STAT3. EB selectively inhibited constitutive as well as IL-6-induced phosphorylation of STAT3 and induced apoptosis of STAT3-dependent tumor cells. EB did not affect the upstream protein tyrosine kinases or the phosphatase (PTPase) of STAT3, but rather interacted directly with STAT3. The effects of EB could be abolished by DTT or GSH, suggesting a thiol-mediated covalent linkage between EB and STAT3. Site mutagenesis of cysteine in and near the SH2 domain of STAT3 identified Cys712 to be the critical amino acid for the EB-induced inactivation of STAT3. Furthermore, LC/MS/MS analyses demonstrated that an α, β-unsaturated carbonyl of EB covalently interacted with the Cys712 of STAT3. Computational modeling analyses also supported a direct interaction between EB and the Cys712 of STAT3. These data strongly suggest that EB directly targets STAT3 through a covalent linkage to inhibit the phosphorylation and activation of STAT3 and induces apoptosis of STAT3-dependent tumor cells.

## Introduction

Signal transducer and activator of transcription 3, known as STAT3, is a transcription factor as well as a signal transducer. In response to cytokines, such as IL-6, and growth factors, such as EGF and IGF, STAT3 is recruited from the cytosol to associate with the activated receptors through its phosphor-tyrosine recognition SH2 domain, and phosphorylated on its carboxy-terminal tyrosine (Tyr705) and serine (Ser727) by the receptor-associated JAK kinases, Src, or other kinases. The tyrosine 705- phosphorylated STAT3 then dimerizes and translocates into the nucleus, where it binds to specific promoter sequences and regulates the expression of target genes, such as cyclin D1, bcl-XL, and c-myc, that are involved in cell growth and survival [1–3]. The serine 727-phosphorylated STAT3, on the other hand, is localized in mitochondria, regulating metabolic functions in mitochondria and supporting the Ras-mediated malignant transformation [4–6]. Aberrant activation of STAT3 has been found in many cancer cells, which contributes to carcinogenesis and tumor progression by promoting cell survival and growth [7–10].

Because of the importance of STAT3 in regulating cell growth and survival, the STAT3 signaling pathway has been considered as a valid target for anti-cancer drugs [11,12]. A number of STAT3 signaling pathway inhibitors have been discovered, most of which are inhibitors for the upstream kinases of STAT3, particularly JAK2, and are not STAT3 pathway-specific [13,14]. Others, such as Stattic [15], cryptotanshinone [16], and S3I-201 [17], target STAT3 directly, but few of them are currently in clinical trials and none of them has become clinical drugs. Therefore, more STAT3 pathway-specific inhibitors are needed for developing novel anti-cancer drugs.

To identify new STAT3 pathway inhibitors, we screened a traditional Chinese herb medicine compound library and found Eriocalyxin B (EB) as a potent and specific STAT3 pathway inhibitor. EB is a natural diterpenoid from Isodon eriocalyx var. laxiflora of the Labiatae family which has been reported to possess various bioactivities, especially anti-cancer, anti-inflammation, and anti-bacteria activities [18]. EB has been reported to induce apoptosis of leukemia cells in vitro and in vivo [19,20]. Structurally, EB belongs to 7, 20-epoxy-ent-kaurane-type diterpenoid and contains two α, β-unsaturated carbonyls that are chemically active electrophiles [21] and are critical for its biological activities.

In the present report, we studied the molecular mechanisms of the selective inhibition of STAT3 by EB. We found that EB specifically inhibited STAT3 activation by covalently binding to the Cys712 near the SH2 domain of STAT3 through a Michael addition with its α, β-unsaturated carbonyl and prevented it to be phosphorylated and activated by its upstream kinases. This study uncovered a new strategy to specifically inhibit the STAT3-mediated signaling and provided a novel STAT3 inhibitor for potential cancer treatment.

## Materials and Methods

### Chemicals and reagents

The Chinese medicinal herb compound library was a collection of 182 natural compounds isolated from 69 traditional Chinese medicinal herbs (unpublished results). The compounds were dissolved in DMSO at a concentration of 10 mM. The final concentration of a compound in the screening assay was 10 μM. The library screening was performed using a cellular luciferase gene reporter assay as described below. EB (>98% purity) was purchased from Shanghai Boylechem Co.ltD. Stattic was purchased from Selleck Chemicals. Sodium orthovanadate and DAPI (Diamidino-phenyl-indole) were purchased from SIGMA. Antibodies against STAT3, p-STAT3 Tyr705, p-STAT3 Ser727, STAT1, p-STAT1 Tyr701, STAT5, p-STAT5 Tyr694, JAK2, p-JAK2 Tyr1007/1008, JAK1, p-JAK1 Tyr1022/1023, Tyk2, p-Tyk2 Tyr1054/1055, PARP and actin were purchased from CST (Cell Signaling Technology). Antibodies against tubulin were purchased from Santa Cruz Biotechnology. Secondary HRP-conjugated antibody was purchased from MultiSciences Biotech. Alexa Fluor 488 donkey anti-mouse IgG antibody was purchased from Invitrogen.

### Cell lines and culture

The HepG2/STAT3 luciferase gene reporter cell line was a gift from Prof. Xinyuan Fu (National University of Singapore), and was cultured in Minimal Essential Medium with 10% FBS. The cell line was generated by stably transfecting the HepG2 cells with a STAT3-responsive firefly luciferase reporter plasmid and was authenticated using the IL-6-induced STAT3 responsive luciferase reporter assay within 6 months. MDA-MB-231, MDA-MB-468, and MDA-MB-453 cells, which were cultured in DMEM with 10% FBS, and A549 cells, which were cultured in RPMI 1640 with 10% FBS, were all obtained from American Type Culture Collection (ATCC). They were passaged for less than 6 months and maintained as recommended by the provider. ATCC performed cell line authentication using DNA fingerprinting by short tandem repeat (STR) analysis.

### Luciferase assay

The HepG2/STAT3 luciferase reporter cells (3.0*10^5^ per well) were seeded into 12 well cell culture plates (Corning) and allowed to grow for 24 h and then treated with EB for 2 h followed by stimulation with IL-6 (50 ng/mL) for 5 h. Luciferase activity was determined using the Promega luciferase kits according to the manufacturer’s instruction. The cell number was counted at seeding and equal number of cells was seeded. All luciferase assay experiments were performed in triplicates to minimize the difference caused by cell number.

### Western blot analysis

Cells were lysed in 1X Laemmli sample buffer (Sigma-Adrich). The extracts were heated at 100°C for 5 minutes and proteins were separated by 8% SDS-PAGE, transferred to a nitrocellulose membrane (Whatman) and probed first with specific primary antibodies, then with horseradish peroxidase-conjugated secondary antibodies. The membranes were developed with enhanced chemiluminescence (ECL) detection system (Thermo).

### Electrophoretic Mobility Shift Analysis

A549 cells were cultured in serum-free medium for 12 h, and then stimulated with IL-6 (20 ng/ml) for 40 min. The nuclear lysates were prepared essentially as described by Chaturvedi MM et al [22]. To test the inhibition of EB on STAT3 DNA-binding, 3 μL of nuclear protein extract (approximately 2 μg/μL) was incubated with different concentrations of EB dissolved in DMSO at 37°C for 1 h, and then was added to 2 μL binding buffer (65 mM HEPES [pH 7.8], 0.5 mM EDTA, 40% glycerol) and 1 μg poly(dI-dC). For competition binding assays, unlabeled oligonucleotide was added to the reaction in 2000-fold molar excess. For the super-shift control, 0.1 mg anti-STAT3 antibody was added to the reaction mixtures. The total reaction volume was 20 μL. All reaction mixtures were incubated at room temperature for 30 min, prior to addition of the biotin-labeled oligonucleotides (upper strand sequence: 5-AGCTTCATTTCCCGTAAATCCCTA-3) for 30 min at room temperature. Protein-DNA complexes were resolved on a 4.5% native acrylamide gel, then transferred to nylon membranes (Whatman), and cross-linked for 10 min under a hand-held UV lamp equipped with a 254-nm bulb. Cross-linked biotin-labeled DNA was probed with horseradish peroxidase-conjugated streptavidin and was detected using the enhanced chemiluminescence (ECL) detection system (Thermo).

### Immunofluorescence Assay

A549 cells were cultured on coverslips in serum-free 1640 medium for 24 h. After incubation with or without 10μM EB for 2 h, cells were stimulated with 50 ng/ml IL-6 for 30 min. Cells were fixed with 4% paraformaldehyde for 10 min and permeabilized for 10 min with 1% Triton X-100 in PBS. After blocking with 1% donkey serum, the coverslips were incubated with a primary antibody against STAT3 for 1 h, followed by incubation with a fluorescence-labeled secondary antibody for 1 h. Cells were counterstained with DAPI. The samples were imaged by confocal microscopy at 20× magnification.

### In vitro kinase assay

Human JAK2, cSRC, EGFR, IKKβ, MAPK1 and PI3K in vitro kinase assays were performed by Eurofins' KinaseProfiler service (http://www.eurofins.com/pharma-services/pharma-discovery-services/services/in-vitro-pharmacology/kinases/biochemical.aspx).

### MTT assay

Cells (3,000/well) were seeded into 96-well plates. Twenty-four hours later, cells were treated with vehicle control (DMSO) or EB (0.1, 0.5, 1, 5, 10, 20 μM) for 48 h. 3-(4, 5-Dimethylthiazol-2-yl)-2, 5-diphenyltetrazolium bromide solution was then added to each well. After 5 h of incubation at 37°C, extraction buffer (10% SDS, 5% isobutanol, and 10 mM hydrochloric acid) was added. The cells were incubated overnight at 37°C, and the absorbance was measured at 595 nm using Tecan Infinite 200 Pro microplate reader (Tecan).

### Site mutagenesis and transfection

Myc-tagged STAT3 plasmid was a gift from Eugene Chin’s lab (Surgery Department of Rhode Island Hospital, USA). Cys to Ser mutations of C418S, C426S, C468S, C542S, C550S, C687S, C712S, C718S STAT3 mutants were generated by Fast Mutagenesis System (TransGen Biotech) and verified by DNA sequencing (Invitrogen). Transient transfection of A549 cells was performed using Lipofect 2000 (Invitrogen) according to the manufacturer’s instructions.

### HPLC-MS analysis

EB was incubated with GSH, FT-8 (FICVTPTT) or QP-10 (QFTKCCPPKP) peptides at 37°C for 2 h. Then the samples were injected in the liquid chromatography-mass spectrometry (LC-MS) system using methanol/water (1:1) as the mobile phase. HPLC analysis was performed using an Angilent HP 1100 series HPLC system (Angilent Technologies). Mass spectrometry was recorded using a LCQ Deca ion trap mass spectrometer (ThermoFinnigan).

### HPLC–MS/MS analysis

EB was incubated with the FT-8 (FICVTPTT) peptide at 37°C for 2 h, then vacuum-dried. The dried peptide samples were dissolved in 4-μl HPLC buffer A (0.1% formic acid in water, v/v) and delivered onto the capillary RPLC trap column (100 μm inner diameter x2 cm, packed with Luna C18, 5 μm, 100 Å pore size, Dikma, China.). After loading and washing, the peptides were transferred to analytical column (10 cm length with 75 μm ID) and were eluted with a 10min gradient of 2% to 80% HPLC buffer B (0.1% formic acid in acetonitrile, v/v) in buffer A at a flow rate of 300 nl min^−1^. The eluted peptides were ionized and introduced into an LTQ Orbitrap Elite mass spectrometer using a nanospray source. Survey full-scan MS spectra (from m/z 350–1,500) were acquired in the Orbitrap with resolution R = 24,000 at m/z 400. The 20 most intense ions were sequentially isolated in the linear ion trap and subjected to collisionally induced dissociation (CID) with a normalized energy of 35%. The exclusion duration for the data-dependant scan was 60 sec and the repeat count was 2.

### Tryptic digestion and LC-MS/MS analysis

Bacteria-expressed STAT3 protein (0.2 mg/ml) (ProteinOne, Inc.) was treated with EB (20 μM) at 37°C for 2 h, and the sample was then separated by SDS-PAGE and stained with Coomassie blue. Coomassie-stained band was excised from the gel and cleaned by dH2O water, and then was digested by trypsin at 37°C overnight. After being vacuum-dried, The samples were dissolved in 4-μl HPLC buffer A (0.1% formic acid in water, v/v) and delivered onto the capillary RPLC trap column (100 μm inner diameter x2 cm, packed with Luna C18, 5 μm, 100 Å pore size, Dikma, China.). After loading and washing, the samples were transferred to analytical column (10 cm length with 75 μm ID) packed with C18resin (3-μm particle size, 90-Å pore size, Dikma, China) connected to an EASY-nLC 1000 HPLC system and were eluted with a 110 min gradient of 5% to 80% HPLC buffer B (0.1% formic acid in acetonitrile, v/v) in buffer A at a flow rate of 200 nl min^−1^. The eluted samples were ionized and introduced into an LTQ Orbitrap Elite mass spectrometer using a nanospray source. Survey full-scan MS spectra (from m/z 320–1,800) were acquired in the Orbitrap with resolution R = 24,000 at m/z 400. The 15 most intense ions were sequentially isolated in the linear ion trap and subjected to collisionally induced dissociation (CID) with a normalized energy of 35%. The exclusion duration for the data-dependant scan was 30 sec and the repeat count was 2.

### Molecular Docking

The structure of Eriocalyxin B was drawn using Maestro 9.0 and prepared using LigPrep. Epik option was employed to predict the putative ionization states at physiological condition, and conformers were generated using the MMFF force field. The crystallographic structure of STAT3 (PDB entry:1BG1) was downloaded from RCSB Protein Data Bank (http://www.rcsb.org/pdb/home/home.do), which was then imported to Maestro and processed using Protein Prepare Wizard. Water was removed and global energy was minimized until RMSD value was below 0.3 angstrom using OPLS-AA force field. The grid box centered at Cys712 near the SH2 domain of STAT3 was generated using Glide, and the standard precision mode was utilized during the docking simulation. The top scored pose was extracted and visualized using Pymol.

### xCELLigence Real-Time Cell Analysis (RTCA)

Cell proliferation was measured using the RTCA system (Roche). Initially, 50 μL of cell free growth medium (10% FBS) was added to the wells. After leaving the devices at room temperature for 30 min, the background impedance for each well was measured. Then 50 μL of the A549 cell suspension was seeded into the 16 well E-Plate (3000/well). After leaving the plates at room temperature for 30 min to allow cell attachment, they were locked in the RTCA DP device in the incubator for 24 h. After 24 h of RTCA profiling, the assay was paused, and the E-Plate were removed from the RTCA system. The existing media was carefully removed and replaced with the media containing IFN-γ (10000 U), EB (1 μM), or their mixture. The E-Plate 16 was placed back into the RTCA system and the cell growth was monitored for 48 h. CI (Cell index) was monitored every 5 min during the experiment. Results were plotted using the RTCA software.

## Results

### EB specifically inhibited the cytokine-induced as well as the constitutive STAT3 activation

**Fig 1:**
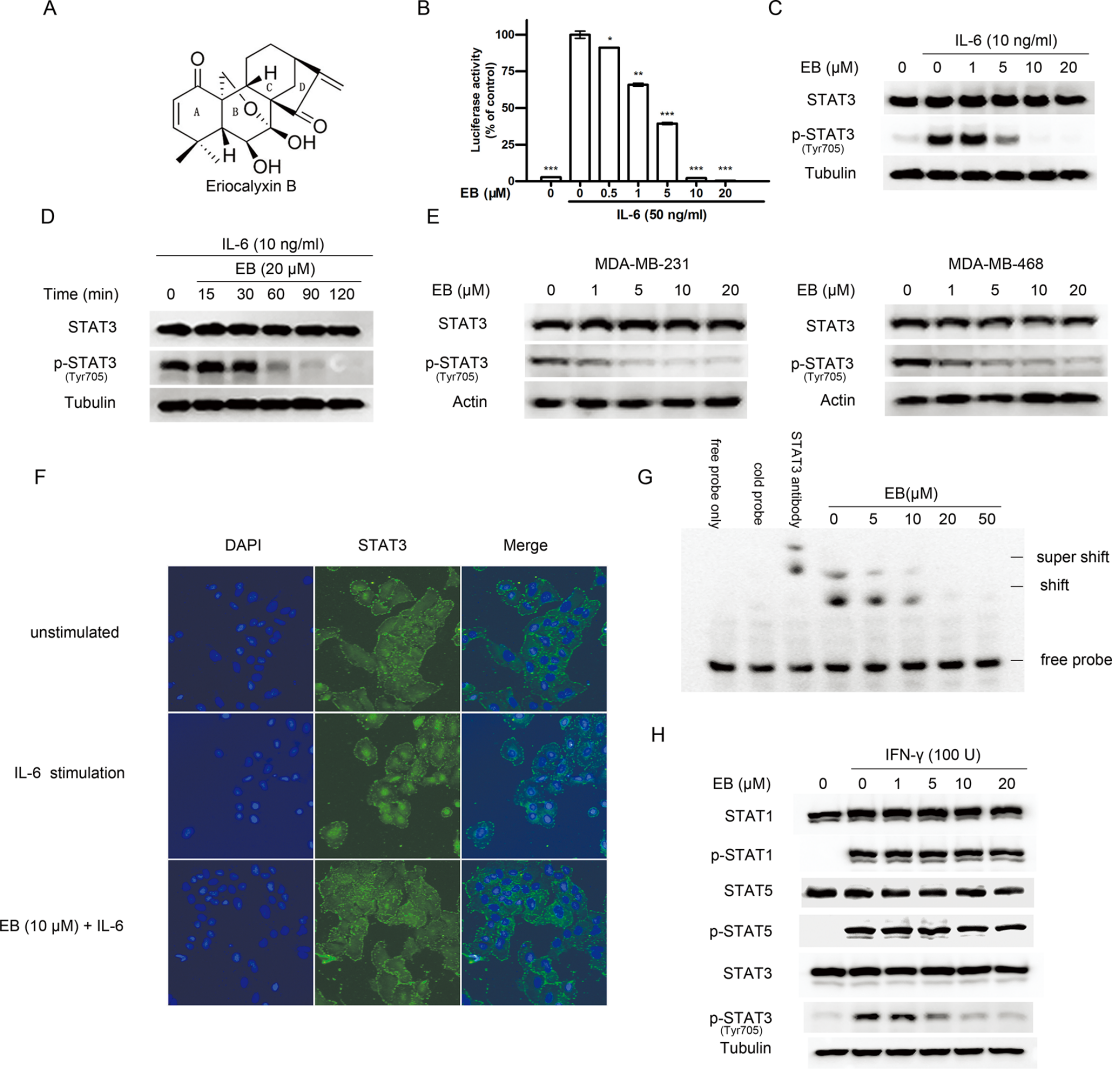
EB specifically inhibited the activation of STAT3. **(A)** Chemical structure of EB. **(B)** HepG2/STAT3-luceferase reporter cells were pretreated with EB at indicated concentrations for 2 h, and luciferase activity was measured following stimulation with IL-6 (50 ng/ml) for 5 h. Data are expressed as mean ± SD. *P < 0.05, **P < 0.01 and ***P < 0.001 versus the control group without EB but with IL-6 stimulation. **(C)** A549 cells were pretreated with EB at indicated concentrations for 2 h before stimulation by IL-6 (10 ng/ml) for 15 min. Whole cell lysates were processed for western blot analysis with the indicated antibodies. **(D)** A549 cells were pretreated with 20 μM EB for various time periods (0–120 min) before stimulation by IL-6 for 15 min. Whole cell lysates were processed for western blot analysis with the indicated antibodies. **(E)** MDA-MB-231 and MDA-MB-468 cells were treated with EB at indicated concentrations for 2 h. Whole cell lysates were processed for western blot analysis with indicated antibodies. **(F)** A549 cells were cultured on coverslips in serum-free medium for 24 h. The cells were then pretreated with vehicle or 10 μM EB for 2 h, followed by 30 min stimulation with IL-6. The cells on the coverslips were then processed for immunochemical staining with an anti-STAT3 antibody or nuclei staining with Diamidino-phenyl-indole. (**G**) EB and nuclear extract from A549 cells that contained activated STAT3 proteins were preincubated for 1h prior to addition of DNA probe. The STAT3 DNA-binding activity was assessed by EMSA. The oligo band shift caused by STAT3 binding and the super shift caused by the anti-STAT3 antibody binding are indicated. **(H)** A549 cells were treated with EB at indicated concentrations for 2 h, followed by stimulation with IFN-γ for 15 min. Whole cell lysates were processed for western blot analysis using the antibodies as indicated.

To identify novel and specific inhibitors of STAT3, we screened a traditional Chinese medicinal herb compound library using a STAT3 responsive luciferase reporter assay system [23]. We identified EB as a strong inhibitor of the IL-6-induced STAT3-responsive luciferase activity in a dose-dependent manner (Fig 1A and 1B).

To verify whether EB inhibited STAT3 activation, we examined the phosphorylation of STAT3 in lung adenocarcinoma A549 cells using Western Blot analysis. We found that EB inhibited the IL-6-induced STAT3 Tyr705 phosphorylation in a dose and time-dependent fashion (Fig 1C and 1D). EB also had similar inhibitory effects on the constitutively Tyr705-phosphorylated STAT3 in breast cancer MDA-MB-231 and MDA-MB-468 cells [24] (Fig 1E). However, EB did not inhibit the IL-6-induced Ser727 phosphorylation of STAT3 (S1 Fig), suggesting that EB inhibited STAT3 activation through inhibiting Tyr705 phosphorylation of STAT3.

Phosphorylation of STAT3 on Tyr705 results in homo- or hetero-dimerization of STAT3, enabling its nuclear translocation and DNA binding [1]. To further confirm the inhibition of EB on STAT3 activity, we investigated the effects of EB on STAT3 nuclear translocation and DNA binding. As shown in Fig 1F and 1G, EB effectively inhibited the IL-6-induced nuclear translocation of STAT3 in the A549 cells, and also inhibited STAT3 DNA-binding activity in a dose-dependent fashion in the in vitro EMSA analysis.

To understand the nature of the inhibition of STAT3 signaling by EB, we analyzed the effects of EB on the signaling of its close family members STAT1 and STAT5, which are structurally similar to STAT3, but have different functions in regulating cell growth [25,26]. Similar to the inhibition of the IL-6-induced STAT3 phosphorylation, EB also inhibited the IFN-γ-induced STAT3 phosphorylation, but had no effects on the IFN-γ-induced STAT1 and STAT5 phosphorylation, suggesting that EB inhibited STAT3 phosphorylation specifically (Fig 1H).

### EB did not inhibit the upstream components of STAT3 activation

**Fig 2:**
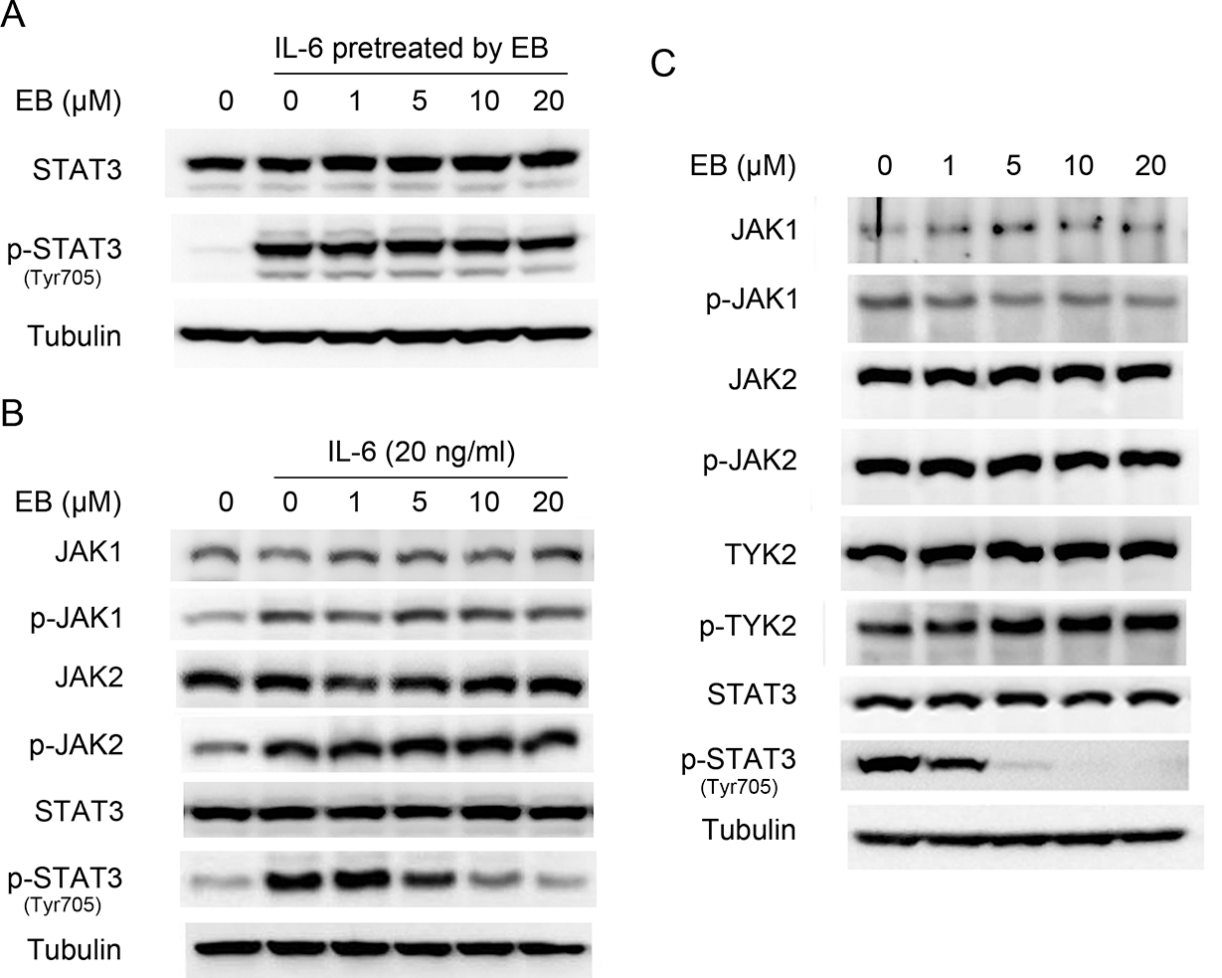
EB did not inhibit the upstream components of STAT3 signaling. **(A)** IL-6 (10 ng/ml) was pretreated with EB at indicated concentrations in vitro for 30 min, and then was used to stimulate A549 cells for 15 min. Whole cell lysates were processed for western blot analysis with the indicated antibodies. **(B)** A549 cells were serum starved for 24 h, and then treated with EB at indicated concentrations for 2 h before stimulation with IL-6 for 15 min. Whole cell lysates were analyzed by western blot using the indicated antibodies. **(C)** A549 cells were pretreated with indicated concentrations of EB for 2 h, whole cell lysates were processed for western blot analysis using antibodies as indicated.

Binding of IL-6 to its receptor initiates cellular events including activation of STAT3 upstream kinases, which then phosphorylate and activate STAT3 [27]. We examined whether EB might interact with IL-6 directly, thereby abrogating STAT3 activation. However, pretreatment of IL-6 with EB did not affect the IL-6-induced STAT3 phosphorylation, suggesting that EB did not interact with IL-6 directly (Fig 2A).

JAK family kinases, particularly JAK1, are the upstream kinases of STAT3 [28]. To investigate whether the inhibition of STAT3 activation by EB was a consequence of the inhibition of its upstream kinases, the effects of EB on the phosphorylation of JAK1, JAK2, and TYK2 were analyzed. As shown in Fig 2B, EB inhibited the IL-6-induced phosphorylation of STAT3 completely at 20 μM, but had no effects on the IL-6-induced phosphorylation of JAK1 and JAK2, implying that EB did not affect the upstream components of the JAKs (Fig 2B). Similarly, EB had no effects on the constitutive phosphorylation of JAK1, JAK2, or TYK2 (Fig 2C and S2 Fig).

To further verify our observation, we analyzed the effects of EB on JAK2 and other kinases in the in vitro kinase assays. EB did not inhibit the activities of any of the STAT3 kinases, such as JAK2, EGFR, cSrc [29], or the STAT3-unrelated kinases, such as MAPK1 or PI3K [Table 1]. Therefore, EB did not inhibit the STAT3 Tyr705 phosphorylation through inhibition of its upstream components.

**Table 1:** Effects of EB (50 μM) on in vitro kinase activities.

Kinase	cSRC (h)	EGFR (h)	IKKβ (h)	JAK2 (h)	MAPK1 (h)	PI3Kinase (h)
Activity (%)	102	118	66	103	93	101

The kinase assay was performed as described in the Methods.

### EB did not affect the dephosphorylation of STAT3

**Fig 3:**
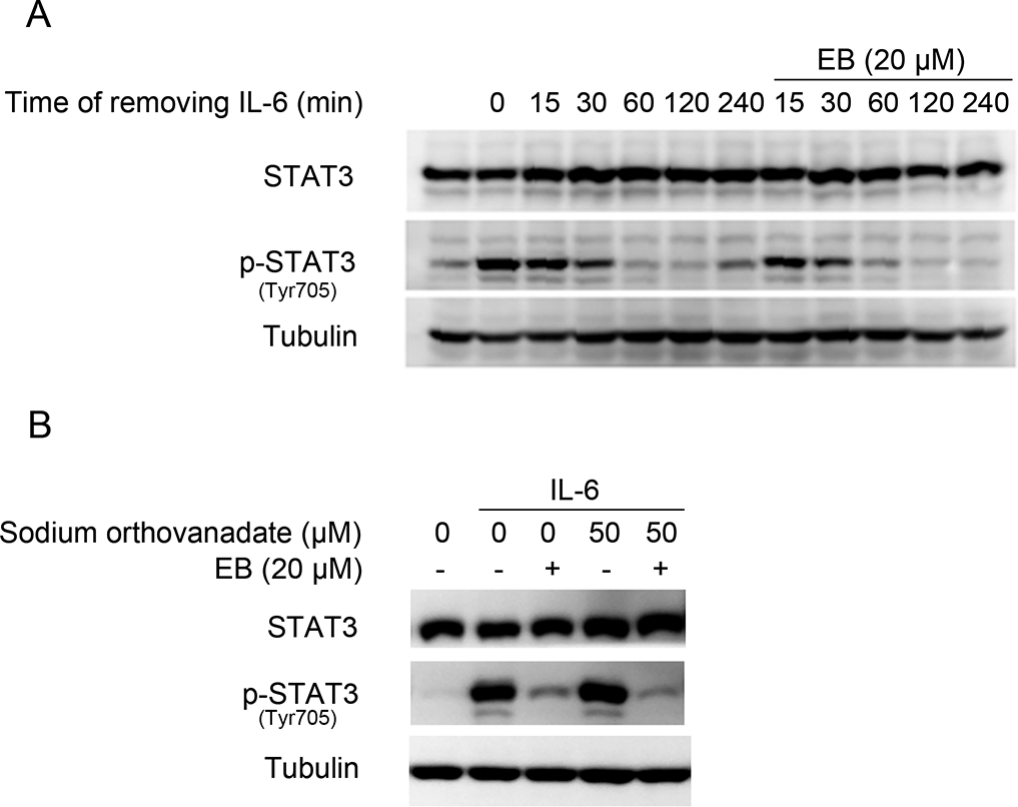
EB did not enhance the activity of tyrosine phosphatases. **(A)** A549 cells were stimulated by IL-6 (10 ng/ml) for 15 min. The media were then replaced by fresh media without IL-6 and incubated with or without EB (20 μM) for different time periods (0–240 min). Whole cell lysates were processed for western blot analysis using antibodies as indicated. **(B)** A549 cells were pre-treated with sodium orthovanadate (50 μM), EB (20 μM) or their mixture for 2 h, then stimulated with IL-6 for 15 min. Whole cell lysates were processed for western blot analysis using antibodies as indicated.

Because protein tyrosine phosphatases (PTPs) are also involved in the regulation of STAT3 phosphorylation [30], we investigated whether the EB-caused inhibition of STAT3 phosphorylation was due to activation of PTPs. The phosphorylation of STAT3 in A549 cells was pulse-stimulated with IL-6 for 15 minutes, followed by a chase in replaced medium without IL-6 for different time periods and the phosphorylation of STAT3 was analyzed. The IL-6-induced STAT3 phosphorylation in A549 cells was transient and the dephosphorylation occurred immediately after stimulation and was completed at 1 hour. Addition of EB did not affect the dephosphorylation of STAT3, suggesting that EB had no effects on the phosphatase activities (Fig 3A). In addition, PTP inhibitor sodium orthovanadate did not block the EB-caused inhibition of STAT3 phosphorylation (Fig 3B). These results demonstrated that EB did not inhibit the STAT3 phosphorylation through enhancing the activities of PTPs.

### DTT and GSH abolished the inhibition of STAT3 phosphorylation by EB

**Fig 4:**
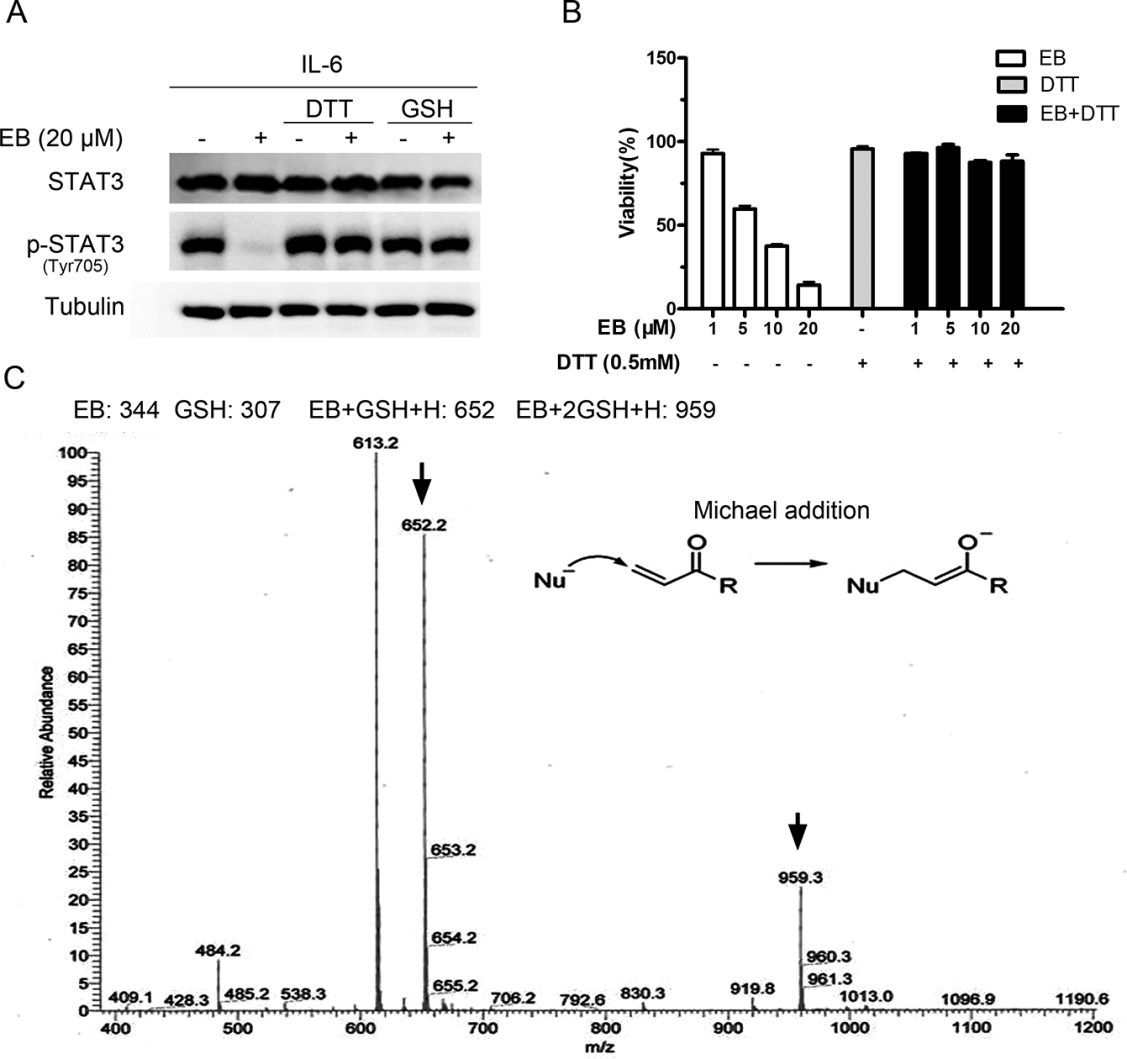
DTT and GSH abolished the EB-caused JAK-STAT3 inhibition. **(A)** A549 cells were pretreated with DTT (0.5 mM), GSH (2 mM), EB (20 μM) or their mixture (EB and DTT/GSH were pre-incubated at RT for 30 min) for 2 h and then stimulated with IL-6 for 15 min. Whole cell lysates were processed for western blot analysis using antibodies as indicated. **(B)** A549 cells were cultured in the presence of indicated concentrations of EB or EB + DTT (0.5 mM) for 48 h and then the viability of A549 cells was analyzed by MTT assay. **(C)** EB (50 μM) was incubated with GSH (250 μM) at 37°C for 2 h, and the products were resolved by mass spectrometry. A possible Michael addition reaction between EB and GSH is illustrated. The black arrows indicated the molecular weights of EB+GSH and EB+2GSH.

EB contains two α, β-unsaturated carbonyl groups which have potential to react with thiols of cysteine and are critical for its biological activities [21]. To investigate a possible covalent interaction between the α, β-unsaturated carbonyl groups of EB and the cysteine of STAT3, EB and DTT/GSH were pre-incubated at RT for 30 min and were then added to the cells. DTT or GSH completely abolished the EB-caused inhibition of STAT3 Tyr705 phosphorylation (Fig 4A). The EB-caused inhibition of cell proliferation was also abolished by DTT (Fig 4B).

To further demonstrate a possible covalent interaction of EB with a thiol, EB was incubated with GSH and the products were examined by LC-MS. The LC-MS analysis detected two major products at m/z 652 [EB+GSH+H] and 959 [EB+2GSH+H], indicating the addition of one and two molecules of GSH to one EB respectively (Fig 4C). The possible reaction sites of EB were the two α, β-unsaturated carbonyl groups in the A or/and D rings of EB (Fig 1A).

### The Cys712 of STAT3 was critical for the inhibition of STAT3 by EB

**Fig 5:**
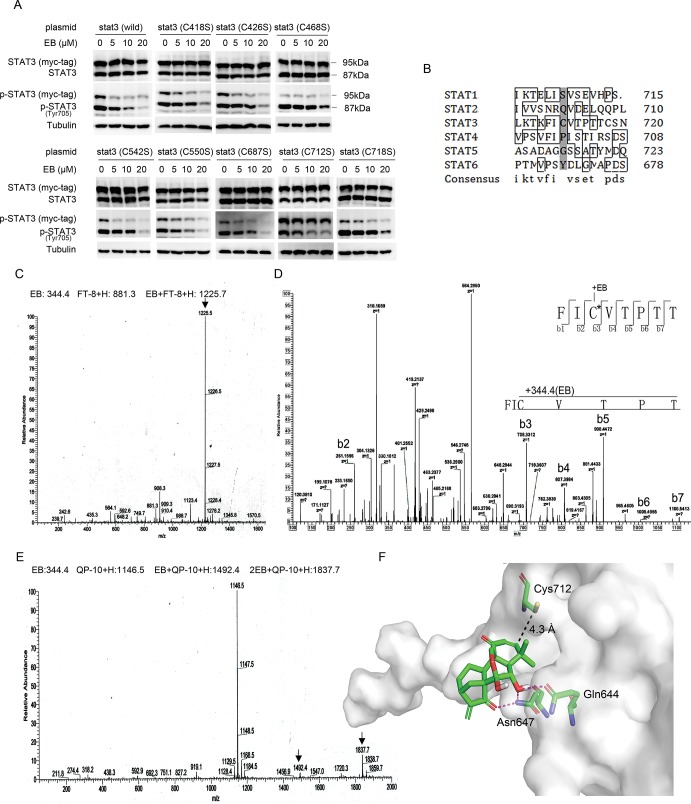
EB covalently targeted STAT3 Cys712. **(A)** A549 cells were transfected with plasmids encoding the myc-tagged wild-type, C418S, C426S, C468S, C542S, C550S, C687S, C712S, or C718S mutation of STAT3 for 24 h. The cells were then treated with EB at indicated concentrations for 2 h before stimulation with IL-6 for 15 min. Whole cell lysates were processed for western blot analysis with anti-STAT3 or anti-p-STAT3 Tyr705 antibodies. The molecular weight of the exogenous myc-tagged STAT3 is 95 kDa, while the endogenous STAT3 is 87 kDa. **(B)** The sequence comparison of all human STAT family members showing that the cysteine at position 712 of STAT3 is unique (highlighted in gray). **(C)** EB (10 mM, 3.75 μL) was incubated with the STAT3 Cys712-containing peptide FT-8 (FICVTPTT) (500 μM, 75 μL) at 37°C for 2 h, and the products were resolved by mass spectrometry. The molecular weight of peptide FT-8 is 881.3 and the molecular weight of the covalent product between EB and FT-8 is 1225.5. **(D)** EB (10 mM, 3.75 μL) was incubated with the peptide FT-8 (FICVTPTT) (500 μM, 75 μL) at 37°C for 2 h, and the products were analyzed by MS/MS. b3, b4, b5, b6, b7 represent the fragmented EB-containing peptides. C* represents the Cys bound by EB. **(E)** EB (20 mM, 3.75 μL) was incubated with peptide QP-10 (QFTKCCPPKP) (500 μM, 75 μL) at 37°C for 2 h, and the products were analyzed by mass spectrometry. **(F)** Computational modeling of the interaction between EB and STAT3. The crystal structure of STAT3 was obtained from PDB (Protein Data Bank). Oxygen atoms of EB were shown in red. Hydrogen bonds between Gln644, Asn647, and EB were shown in purple. The predicted distance between the α, β-unsaturated carbonyl and the thiol of Cys712 is 4.3 À as indicated.

The above results suggested that EB may interact directly with one or more of the cysteines of STAT3 to form a covalent linkage to inhibit STAT3 activity. The STAT3 protein is composed of 6 functional domains [31–34], among which the SH2 domain and the transactivation domain are the most critical regions for STAT3 phosphorylation, dimerization, and activation [35]. To find out which cysteine are likely the sites for EB to attack, we mutated Cys418, Cys426, Cys468, Cys542, Cys550, Cys687, Cys712, and Cys718 in and near the SH2 and transactivation domains of STAT3 to serine respectively and analyzed the effects of the mutations on the EB-caused inhibition of STAT3 phosphorylation. As shown in Fig 5, none of the mutations themselves affected the phosphorylation of STAT3, although the levels of the phosphorylation of the transfected STAT3s were less than that of the endogenous STAT3. EB inhibited the phosphorylation of the endogenous, the transfected wild-type, as well as all of the 7 transfected Cys-mutant STAT3s, except that of the Cys712-mutant STAT3 (Fig 5A). In addition, a sequence comparison between different STATs indicated that the sequences surrounding the Cys712 of STAT3 are not highly conserved. The Cys712 of STAT3 is unique (Fig 5B), which is consistent with the observation that EB had no effects on the IFN-γ-induced STAT1 and STAT5 phosphorylation while inhibited the STAT3 phosphorylation (Fig 1H). These results demonstrated that the Cys712 was critical for mediating the EB inhibition of STAT3 phosphorylation, suggesting that EB might specifically interact with the Cys712 of STAT3 to inhibit the phosphorylation of STAT3.

To demonstrate a direct interaction of EB with the Cys712 of STAT3, we incubated EB with a bacteria-expressed STAT3 protein in vitro. We however could not detect the Cys712-containing peptide of STAT3 in LC-MS analysis. It is possible that the Cys712 of STAT3 may form disulfide bond in vitro to prevent it to be modified by EB and to be detected by mass spectral analysis (S3 Fig). Therefore, we synthesized a peptide, which contains the Cys712 (FT-8: FICVTPTT) of STAT3, and incubated it with EB. A LC-MS analysis of the resulting products identified a product with the molecular weight of 1225.5, which is the molecular weight of the STAT3 peptide plus EB, demonstrating that a covalent linkage has been formed between the STAT3 peptide and EB (Fig 5C). Further LC-MS/MS analysis demonstrated that the covalent linkage was formed between the cysteine and EB (Fig 5D).

To examine the selectivity of EB, we synthesized another cysteine-containing peptide (QP-10: QFTKCCPPKP) and incubated it with EB. The LC-MS analysis showed that its reactivity with EB was 10 times lower than that of FT-8 (Fig 5E), demonstrating the specificity of EB towards the Cys712 of STAT3.

We also performed a computational modeling analysis on the interaction of EB with STAT3. The results of this modeling calculation predicted an interaction between the α, β-unsaturated carbonyl group in the A ring of EB and the Cys712 near the SH2 domain of STAT3 (Fig 5F). The molecular modeling study revealed a stable binding mode for EB in the surface binding pocket of the STAT3 SH2 domain. The Gln644 and Asn647 of STAT3 that make contact with EB form hydrogen bonds with the C6 hydroxyl and the C15 carbonyl group of EB. The C2 and C3 carbons of the α, β-unsaturated carbonyl group in the A ring of EB lie in close proximity to the Cys712 residue in the STAT3, permitting a nucleophilic attack by this thiol group on the electrophilic carbon centers. These data are consistent with and support the results of the site mutagenesis of STAT3.

Taken together, these data provided strong evidences to support the possibility that EB directly interact with STAT3 with a covalent binding to the Cys712 of STAT3 to prevent STAT3 to be phosphorylated.

### EB induced apoptosis of the STAT3-dependent tumor cells

**Fig 6:**
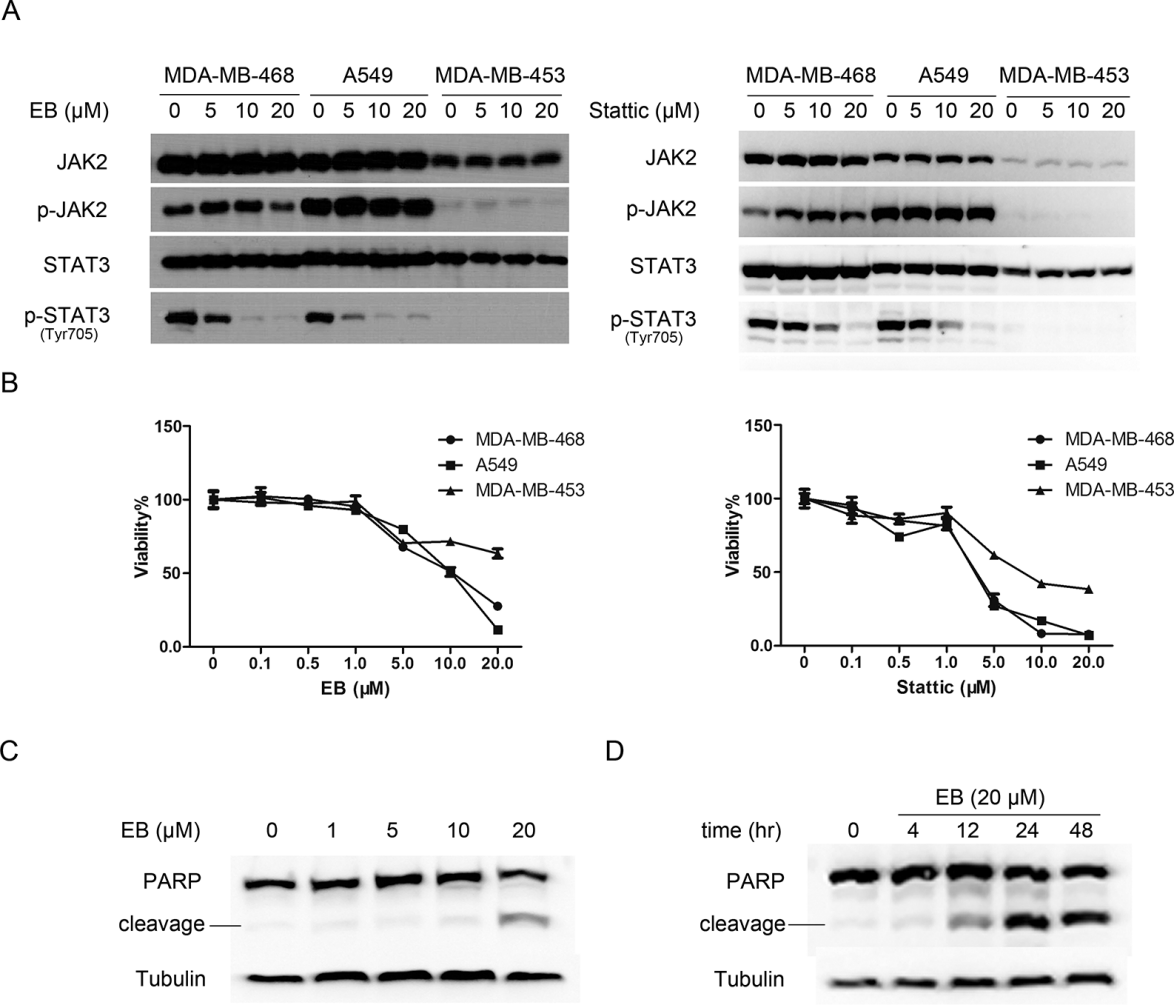
EB inhibited tumor cell growth by inducing apoptosis. **(A)** MDA-MB-468, A549, MDA-MB-453 cells (3.0*10^5^ per well) were treated with EB or Stattic at indicated concentrations for 2 h. Whole cell lysates were processed for western blot analyses using antibodies as indicated. **(B)** MDA-MB-468, A549, MDA-MB-453 cells were treated with EB or Stattic at indicated concentrations for 48 h. The viability of the three cell lines was analyzed by MTT assay. **(C)** A549 cells were treated with EB at indicated concentrations for 12 h. The whole cell lysates were processed for western blot analysis using antibody as indicated. **(D)** A549 cells were treated with EB (20 μM) for various time periods (0–48 h). Whole cell lysates were processed for western blot analysis using antibody as indicated.

STAT3 is constitutively activated and/or overexpressed in many tumor cells including non-small cell carcinoma, breast carcinoma, melanoma, and leukemia cells [36–39], and the inhibition of STAT3 in these cells affects their survival and growth. We examined the effects of EB on tumor cells that had different levels of STAT3 phosphorylation and found that the inhibition of EB on tumor cell growth was correlated with the level of the STAT3 phosphorylation in the cells (Fig 6A and 6B). The A549 and MDA-MB-468 cells, which have relatively high levels of the activated STAT3 and are STAT3-dependent [36,37], were more sensitive to EB than the MDA-MB-453 cells, which had no detectable phosphorylated STAT3 (Fig 6A). We also examined the effects of another STAT3-specific inhibitor, Stattic, which was reported to selectively inhibit the activation of STAT3 [15]. Similar to EB, Stattic also inhibited the growth of A549 and MDA-MB-468 cells more than that of the MDA-MB-453 cells, further indicating that the inhibition of EB on tumor cell growth correlated with the EB-dependent inactivation of STAT3 (Fig 6A and 6B).

To understand the nature of the EB-induced cell growth inhibition, we examined the effects of EB on the induction of apoptosis, as indicated by PARP cleavage, in the A549 cells. We found that EB induced PARP cleavage in A549 cells in a dose and time-dependent fashion (Fig 6C and 6D), indicating that EB inhibited tumor cell growth by inducing apoptosis.

### EB and IFN-γ synergistically inhibited tumor cell growth

**Fig 7:**
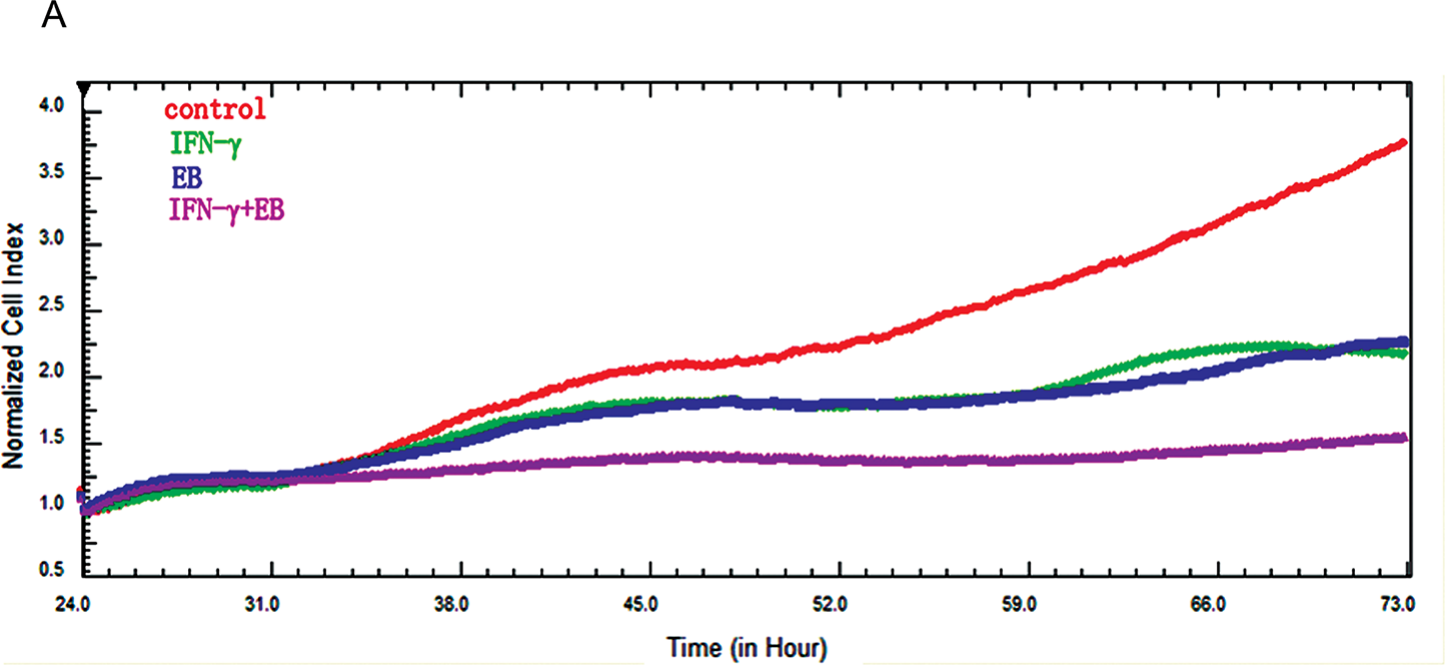
EB and IFN-γ synergistically inhibited tumor cell growth. (**A**) A549 cells were plated into a Real-Time Cell Analyzer (RTCA) for 24 h. IFN-γ (10000 U), EB (1 μM), or their mixture were then added. The cell growth was recorded by the Real-Time Cell Analyzer.

IFN-γ inhibits cell growth by activating STAT1 and its downstream growth inhibitory genes [40]. At the same time, IFN-γ also induces the phosphorylation/activation of STAT3 which promotes cell survival and growth [41]. Because of the apparent opposing effects of STAT1 and STAT3 on cell growth, we tested the possibility of using EB to specifically inhibit STAT3 to enhance the growth inhibitory effects of IFN-γ. Indeed, EB and IFN-γ synergistically inhibited the growth of tumor cells (Fig 7A). These results further confirmed the specificity of EB towards STAT3.

## Discussion

STAT3 is an important signal transducer and transcription factor in regulating genes critical for cell growth and is often activated in tumor cells. Identification of small molecules directly targeting STAT3 has been a well-recognized strategy for treating cancer, particularly the cancer cells with constitutively activated STAT3 [42,43]. Diterpenoids from Isodon species have previously been shown to exert significant cytotoxicity against solid tumor cells as well as hematological malignant cells [18,44]. But the mechanisms by which the diterpenoids induce tumor cell death have not been elucidated. In this study, we identified one of the diterpenoids, EB, as a specific and potent inhibitor of the STAT3 signaling pathway. EB specifically inhibited the cytokine-induced as well as the constitutive STAT3 activation by directly and covalently targeting STAT3.

We presented several lines of evidences to demonstrate that EB covalently bind to the Cys712 of STAT3. First, EB contains two α, β-unsaturated carbonyl groups which are thiol reactive and may form adducts with the thiols of the cysteine in the STAT3 proteins. Second, the inhibition of STAT3 phosphorylation by EB was abolished by DTT or GSH which formed a covalent linkage with EB, possibly through a Michael addition reaction. Third, site mutagenesis of the cysteine in and near the SH2 domain of STAT3 identified the Cys712 to be the critical amino acid for the EB inhibition of STAT3 phosphorylation. Fourth, a Cys712-containing peptide of STAT3 formed a covalent complex with EB. Last but not least, computational modeling analysis indicated that the α, β-unsaturated carbonyl group in the A ring of EB was in close contact with the Cys712 of STAT3 to allow EB to attack the residue to formed a covalent linkage. Taking together, these results strongly support that a Michael addition reaction may have occurred between the α, β-unsaturated carbonyl group of EB and the thiol of Cys712 of STAT3. The covalent modification of STAT3 by EB prevented STAT3 to be phosphorylated and activated, and therefore inhibited STAT3 signaling.

The interaction between EB and STAT3 appeared to be quite specific. EB only inhibited the STAT3 signaling pathway, but not the STAT1 and STAT5 signaling pathways. A sequence analysis and comparison between different STATs indicate that only STAT3 contains the Cys712 (Fig 5B). Therefore, it is the combination of the structure of EB, the sequences surrounding the cysteine712, and the secondary structure of STAT3 that determined the specificity of EB towards STAT3. The specific inhibition of STAT3 is important for treating cancer cells because certain growth suppressive cytokines, such as IFN-γ, activates STAT1, which mediates growth suppressive signals [45], as well as STAT3, which promotes cell growth [46]. Specific inhibition of STAT3 enhanced the growth suppressive activities of the IFN-γ. Therefore, combination of IFN-γ and EB provides a new and better strategy for cancer treatment.

It was previously reported that EB induced apoptosis of tumor cells and differentiation of TH1 and TH17 cells through multiple cellular signaling pathways such as NFκB and MAPK by inducing ROS [19,20,47]. We cannot rule out the possibility that the EB-induced ROS may also play a role in inhibiting STAT3 signaling. However, our data suggest that the direct and specific interaction of EB to STAT3 is the major factor in inhibiting the STAT3 pathway. It is likely that both STAT3 inactivation and ROS contributed to the inhibition of STAT3 signaling and the induction of apoptosis of tumor cells.

There have been several reports demonstrated the in vivo anti-tumor activities of EB [19,48]. The administration of EB dose-dependently inhibited the growth of human pancreatic tumor xenografts in nude mice without significant side effects [48]. EB also induced apoptosis of leukemia cells in vitro and in vivo but did not affect the proliferation capacity of normal hematopoietic progenitor cells [19]. These data, together with ours, strongly demonstrated that EB had great potential to be an anti-cancer drug candidate.

A number of small molecule inhibitors of STAT3 have been reported, including Stattic [15], cryptotanshinone [16], S3I-201 [17], curcubitacins [49,50], withaferin A [51], and C48 [52]. The mechanisms of action of most of the compounds, however, have not been fully understood. What these molecules have in common are reactive chemical moieties and are capable of modifying cysteine residues. We suspect that some of these Michael acceptor-containing compounds may act through alkylation of cysteines of STAT3 as EB does. Indeed, Stattic, which possesses a vinyl sulfone, has been shown to form covalent linkages with a number of cysteines of STAT3 [53], suggesting a none-specific binding to cysteines. C48, on the other hand, forms a covalent linkage specifically with Cys468, another surface-exposed cysteine in the DNA binding domain of STAT3, to block STAT3 to bind to DNA. These observations, together with ours, suggest that covalent binding may be an efficient way to interact with proteins, such as STAT3, which have no deep pockets, or none-pocket regions of a protein to allow high affinity binding for small molecules.

In summary, our study discovered a natural small molecule inhibitor of STAT3, EB, which specifically and covalently binds to Cys712 of STAT3 to inhibit its phosphorylation and activation and induces apoptosis of tumor cells, especially those whose survival and growth are dependent on STAT3. Our study not only uncovered a new compound and a new method to treat cancer, but also introduced a new tool to study the function of STAT3 and a new strategy to inactivate proteins in general.

## Supporting Information

S1 Fig EB did not inhibit the IL-6-induced Ser727 phosphorylation of STAT3. **(A)** A549 cells were pretreated with EB at indicated concentrations for 2 h before stimulation by IL-6 (10 ng/ml) for 15 min. Whole cell lysates were processed for western blot analysis with the indicated antibodies.
(TIF)

S2 Fig EB did not inhibit the constitutively phosphorylated JAK2. **(A)** MDA-MB-231 and MDA-MB-468 cells were treated with EB at indicated concentrations for 2 h. Whole cell lysates were processed for western blot analysis using indicated antibodies.
(TIF)

S3 Fig LC/MS/MS analysis of the EB-treated STAT3 protein. **(A)** EB (20 μM) was incubated with bacteria-expressed STAT3 protein (0.2 mg/ml) at 37°C for 2 h, and the products were separated by SDS-PAGE and digested by trypsin. Tryptic peptides were analyzed by mass spectrometry. The experiment was repeated three times and matched peptides were shown in bold red. **(B)** MS/MS analysis of Cys712 containing peptide (FIC*VTPTTCSNTIDLPMSPR). y3, y5, y6, y7, y8, y9, y10 and y11 are well matched, y12~y18 (between Cys712 and Cys718) are poorly matched, demonstrating a possible disulfide bond between two cysteines. C* represents Cys712.
(TIF)
