# Supplementary figures and images for: Eriocalyxin B Inhibits STAT3 Signaling by Covalently Targeting STAT3 and Blocking Phosphorylation and Activation of STAT3
Source: PLoS One. 2015 May 26;10(5):e0128406. doi: 10.1371/journal.pone.0128406 (PMC4444003; doi:10.1371/journal.pone.0128406)

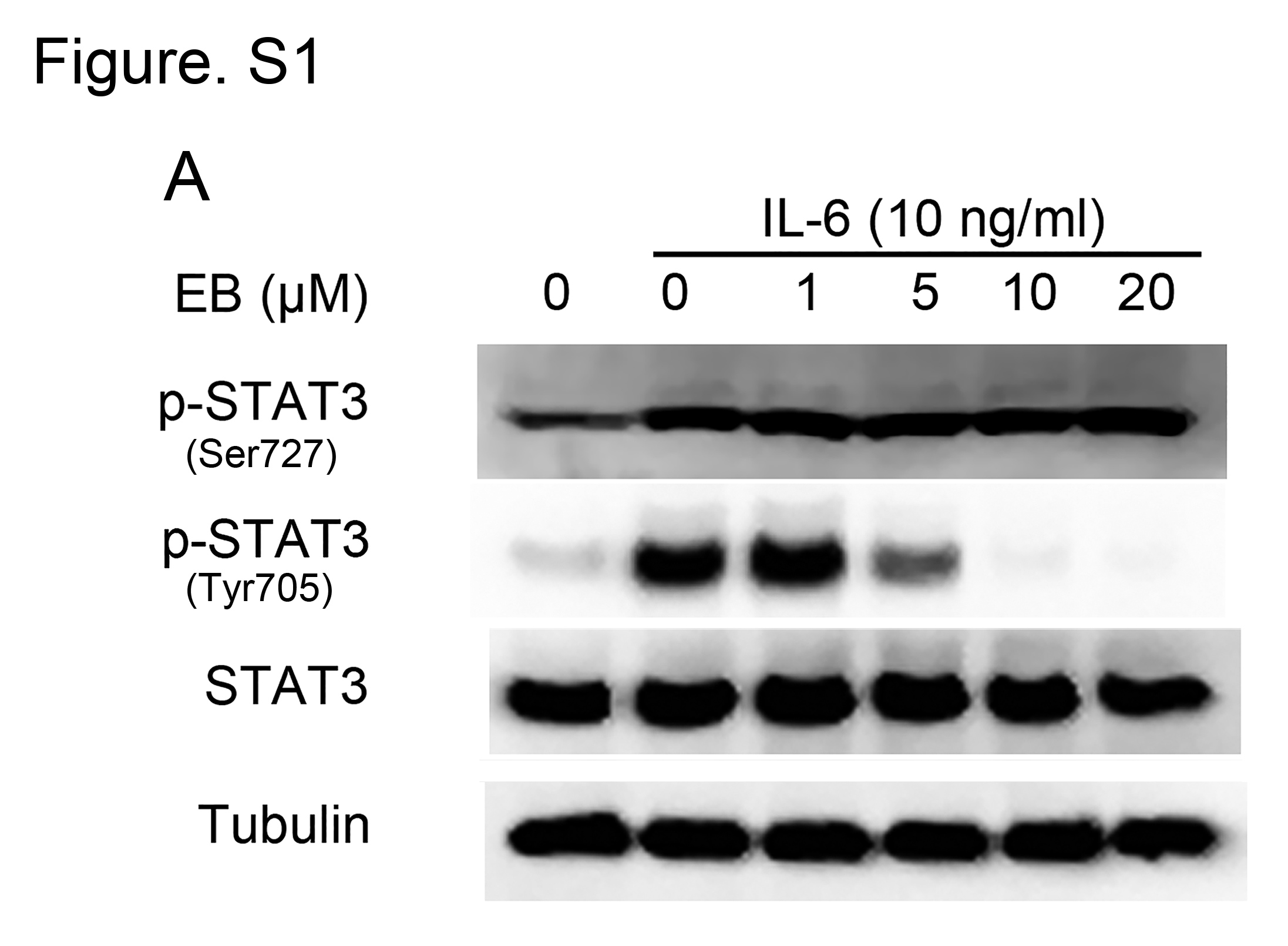

Supplement: S1 Fig — (A) A549 cells were pretreated with EB at indicated concentrations for 2 h before stimulation by IL-6 (10 ng/ml) for 15 min. Whole cell lysates were processed for western blot analysis with the indicated antibodies. (TIF) [file pone.0128406.s001.tif]

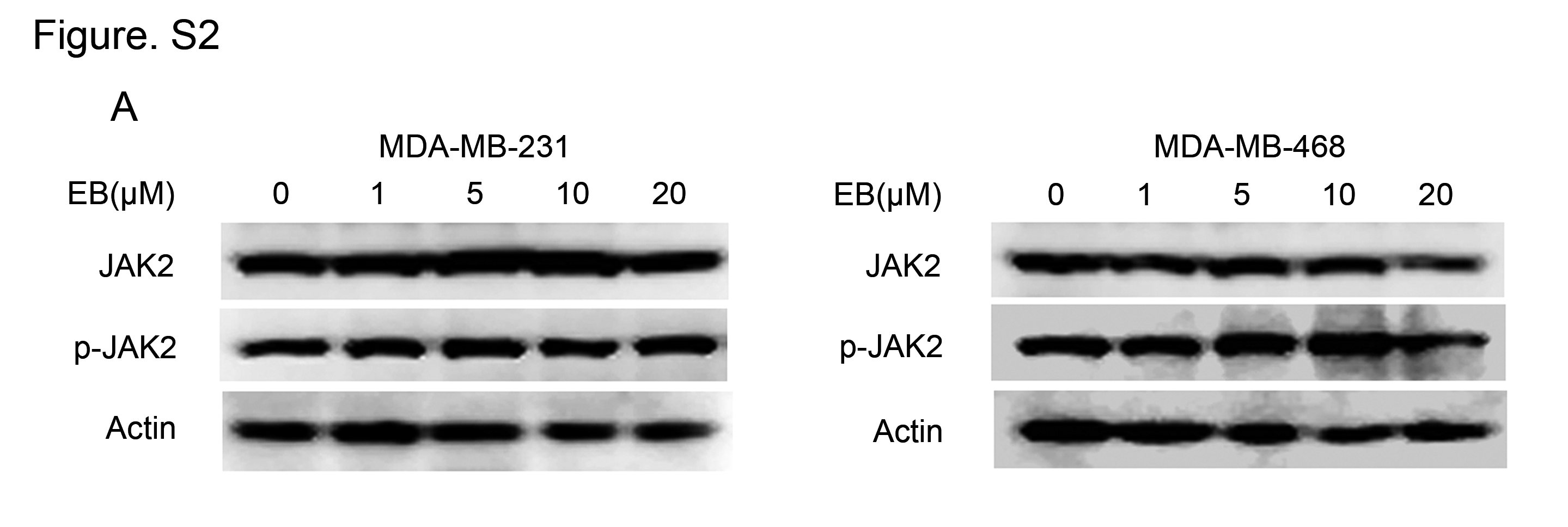

Supplement: S2 Fig — (A) MDA-MB-231 and MDA-MB-468 cells were treated with EB at indicated concentrations for 2 h. Whole cell lysates were processed for western blot analysis using indicated antibodies. (TIF) [file pone.0128406.s002.tif]

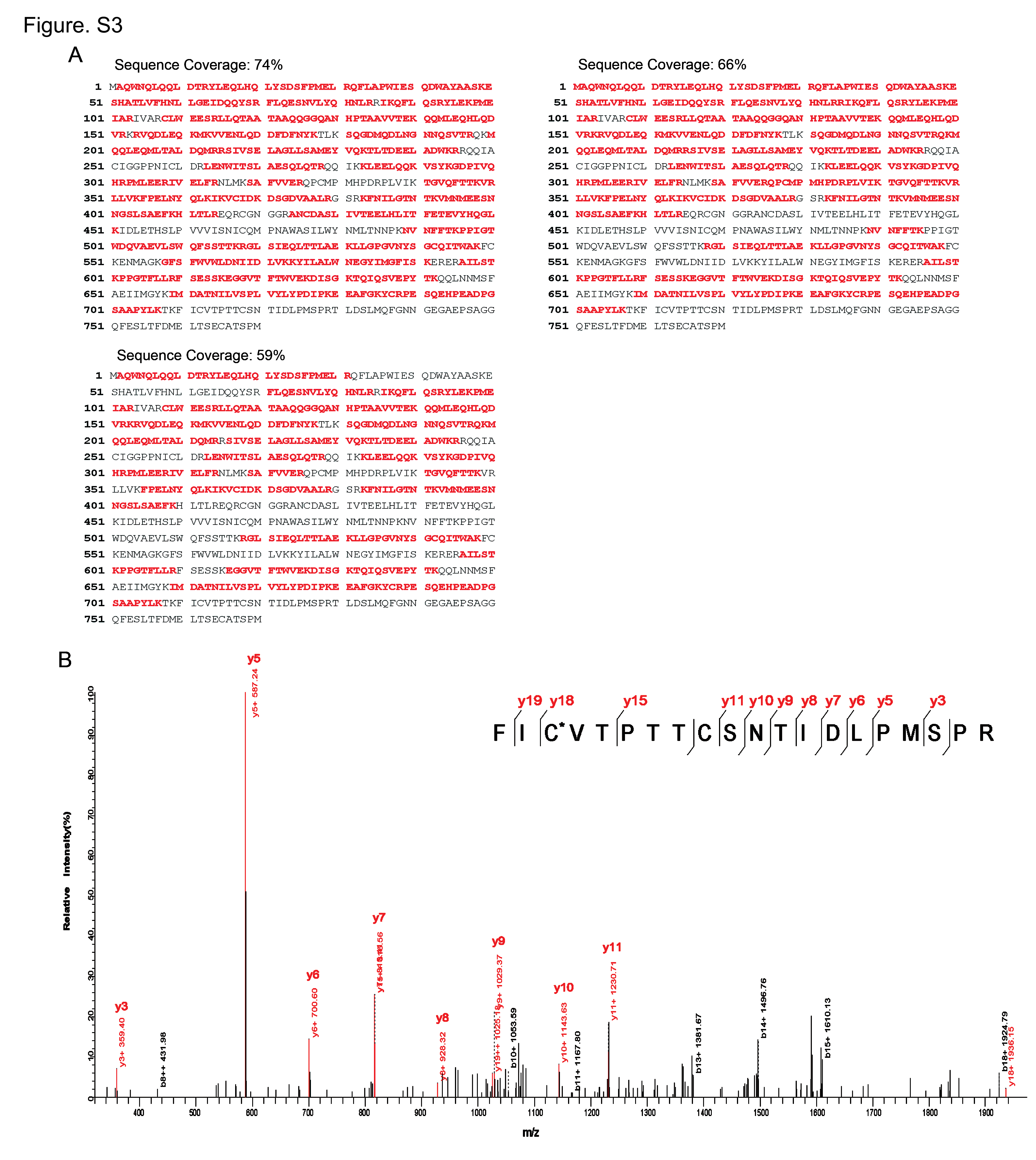

Supplement: S3 Fig — (A) EB (20 μM) was incubated with bacteria-expressed STAT3 protein (0.2 mg/ml) at 37°C for 2 h, and the products were separated by SDS-PAGE and digested by trypsin. Tryptic peptides were analyzed by mass spectrometry. The experiment was repeated three times and matched peptides were shown in bold red. (B) MS/MS analysis of Cys712 containing peptide (FIC*VTPTTCSNTIDLPMSPR). y3, y5, y6, y7, y8, y9, y10 and y11 are well matched, y12~y18 (between Cys712 and Cys718) are poorly matched, demonstrating a possible disulfide bond between two cysteines. C* represents Cys712. (TIF) [file pone.0128406.s003.tif]
